# Impediometric Electrochemical Sensor Based on The Inspiration of Carnation Italian Ringspot Virus Structure to Detect an Attommolar of miR

**DOI:** 10.1038/s41598-020-66393-z

**Published:** 2020-06-15

**Authors:** E. Ghazizadeh, Seyyed Ebrahim Moosavifard, Negin Daneshmand, Saeid kamari Kaverlavani

**Affiliations:** 1 Department of Medical Biotechnology, School of Medicine, Mashhad University of Medical Sciences, Mashhad, Iran; 2 Department of Advanced Medical Sciences & Technologies, School of Medicine, Jahrom University of Medical Sciences, Jahrom, 74148-46199 Iran; 3 Research Center for Noncommunicable Diseases, School of Medicine, Jahrom University of Medical Sciences, Jahrom, 74148-46199 Iran; 4 Department of Materials Science and Engineering, Shiraz university, Shiraz, Iran; 5 Department of Physics, Tarbiat Modares University, Tehran, Iran

**Keywords:** Biophysics, Materials science, Nanoscience and technology, Physics

## Abstract

Electrochemical sensors are the tools to detect the accurate and sensitive miRs. There is the challenge to increase the power and sensitivity of the surface for the electrochemical sensor. We design a virus-like hallow structure of cuco_2_o_4_ that it holds the large amounts of p19 protein by mimicking of inherent virus (Carnation italian ringspot virus) to detect 21mir with the limit of detection (LOD = 1aM). The electrochemical measurements are performed between the potentials at −0.3 V and +0.3 V with 1 mM [Fe(CN)_6_] ^−3/−4^. After dropping the cuco_2_o_4_ on the SCPE (screen carbon printed electrode), the sensor is turned on due to the high electrochemical properties. Then, p19 proteins move into the hallow structure and inhibit the exchange of electrochemical reactions between the shells and the sensor is turned off. Then, adding the duplexes of RNA/miRs cause to increase the electrochemical property of p19 due to the change of p19 conformation and the system is turned on, again. So, for the first time, a virus-like hallow structure has been used to detect the 21miR in the human serum, MCF-7, Hella cells, with high sensitivity, specificity, and reproducibility in few minutes.

## Introduction

MicroRNAs are endogenous, single-stranded, non-coding small RNA with a length of ~22 nucleotide (nt). The alterations in the expression of these molecules have been linked to many diseases and cancers^1^. So, these molecules have been important as diagnostic and prognostic tools in many cancers^2,3^. A wide range of nanoparticles, nanorods, nanowires, nanoclusters, nanocomposites, etc, have been used in the electrochemical biosensor fabrication to detect of miRNA^4,5^. Hollow nanostructures offer promising potential for advanced energy storage and electrochemical applications. The higher weight of the multi-shelled hollow nanostructures can cause to increase the energy density^6,7^. They are designed with a hybrid of internal architectures such as core(s)-in-hollow- shell(s), multishell and multi-chambers^8,9^. P19 protein is a suppressing factor in the silencing pathway as a common form of antiviral defense. This protein is used as a nanotechnology tool for micro RNA quantitation^10,11^. This protein naturally develops from the CIRV (Carnation Italian ringspot virus). Cryo-EM of the CIRV particle showed that they have internal shells with the separates section. The outer surfaces of the shells have a negative charge, and the inner surfaces have a positive charge due to pH-induced morphological changes of proteinaceous viral shells^12,13^. It was demonstrated that p19 and other proteins staying in the virus based on the changes in the charges^14^. There is some research about core-shell structures via virus mimicking for medical applications^15^. In this research; we have constructed the nanomaterials based on the inspiration of the inherent structure of CIRV to use as biosensor applications. This structure consists of a mixture of metals such as cobalt and copper and oxygen (cuco_2_o_4_) which generate shells with positive and negative charges that they can create a strong bed surface. For the first time, these structures have been used in the design of electrochemical sensors to the identification of miR with high sensitivity. After dropping the cuco_2_o_4_ hallow structures on the SCPE-GNP (screen carbon printed electron-gold nanoparticle) electrode, the chemical reaction has increased significantly due to the cobalt/copper-emitting conductive structure with inner shells and outer. Adding the p19 protein to this sensor cause to move the shells via pore by mimicking of CIR virus. These proteins prevent the electrochemical exchange of ions into the shell and outer space, and the sensor resistance is reduced and switched off. Then, we add a duplex of 21miR/RNA which they moved into the shells and connect with p19 tightly. The change in p19 conformation induced more electrochemical reactions and the sensor is on, again. Because of the high p19 content, the sensitivity of this sensor is very high (1 aM). We use this sensor to detect 21miR in a real sample (human serum, MCF-7, Hella cells which they have the differential expression of 21 miR as model cases). So this virus-like sensor can identify the miRs with high sensitivity, reproducibility, and specificity.

## Experimental

All of the chemical materials such as analytical grade potassium ferrocyanide, potassium ferricyanide, sulfuric acid, hydrogen peroxide, sodium chloride, and potassium chloride and cobalt and copper were purchased from chimera co. Glutaraldehyde and Trizol were provided from nano alvand Co. Synthetic oligonucleotides were prepared by MW GE biotech, Ebersberg, Germany. We used as extraction RNA kit and commercially human serum from cinnagen Co (IRAN). The following sequences were used for RNA sensing experiments Probe: 5p′-ACAACAUCAGUCUGAUAAGCUA-3′ (21) miR-21: 5′- UAG CUUAUCAGACUGAUGUUGA -3′ (RNA) mismatch RNA: 5′- GUA GACAAGCAUGACGUACCUG -3′(RNA). P19 was prepared from New England Bio Labs Inc and used without further purification. P19 siRNA Binding Protein (10 units/ml) was stored at −20 °C^16^. Screen-printed carbon electrode modified with gold nanoparticles on the ceramic substrate was purchased from DropSens Inc. This electrode consisted of a GNP-carbon working electrode; a carbon counter electrode and a silver reference electrode.

## Methods

In this study, we used as SP-300 Instruments (SP-300) Texas to detect all of electrochemical analyzes such as electrochemical impedance spectroscopy (EIS) and differential pulse voltammetry (DPV). DPV measurements were done in the presence of 1 mM [Fe(CN)_6_]^−3/−4^ in phosphate Buffer (PS) buffer in the potential window −0.3 V to+0.3 V at a scan rate 50mVs 1. The frequency range used for impedance measurement was 100 kHz to 1 Hz of [Fe (CN) _6_]^−3/−4^ in PBS pH = 7.4^17^. Analysis of EIS spectra was done using equivalent circuits using ZSimpWin 3.22 (Princeton Applied Research) and results reported in Nyquist plots. The AFM and TEM images were gathered using TecnaiG20 instruments from FEI Company and Hillsboro, USA. Particle sizes and zeta potentials were achieved from Horiba nanoparticle size analyzer, Malvern nano SZ-100 which uses green laser light at wavelength 532 nm.

### Preparing cuco2o4 as multi-shelled nanoporous materials

Synthesis of the onion-like nanoporous mixed copper-cobalt oxide hollow spheres was synthesized by a facile self -templating method. In a normal procedure, 0.060 mmol of Co(NO_3_)_2_.6H_2_O and 0.030 mmol of Cu(NO_3_) 2.6H_2_O and 0.08 mmol of isophthalic acid (H_2_IPA) were added to a mixture of 10 mL N,N-dimethylformamide (DMF): acetone (1: 1 v/v) to achieve a clear homogeneous solution by stirring for 6 h. The mixture was transferred into a teflon-lined stainless-steel autoclave and the reaction was kept at 160 C for 4 h^18^. The resultant product was washed by ethanol several times and separated by centrifugation. The Onion-like CuCo_2_O_4_ hollow spheres are generated through thermal treatment in air at the temperature of 500 C for 10 min with a heating rate of 5 C min^−1^.

### Fabrication the sandwiched of cuco2o4/p19/21miR-RNA on the SCPE/GNP

Cuco_2_o_4_ was prepared by dissolving in ethanol at the ratio1:1, dried, rehydrated with buffer (pH = 7.4) and this mixture became a centrifuge at 10,000 rpm for 10 min to prepare supernatant. So, 1.2 µl of this supernatant was dropped on the surface of SCPE/GNP and stored at 4 °C for 1 h. The electrode was dried and carefully rinsed with water, dried again at room temperature, and then used for the electrochemical test. We use p19 to detect the sensitivity of miR. So, we use ten ml of 1:10 (v/v) dilated p19 protein solution were shacked at 37 °C for a 45 min. Then 0.5 µl of p19 was dropped on the cuco_2_o_4_/ SCPE/GNP and incubated at 37 °C for 1 h. To identification the electrochemical behavior of p19 relative to the duplex of miR21-probe, we added the duplex of miR21/RNA. So, 6 mg/ml RNA probe and 3 mg/ml miR21 were blended in a vial containing Tris-EDTA Buffer (TEB). The target was put into the thermal shaker which was set to 65 °C and 250 rpm mixing speed and kept there for 1 h^16^. Then, 1 mM of 2 ml hybrid of miR21 was added on the surface of the electrode and incubated at 37 °C for 2 h in a dark without shaking. Finally, DPV and EIS measurements were carried between the potentials at +0. 3 V and −0.3 V in PBS.

### Identification of the specificity and sensitivity of the cuco_2_o_4_/p19/SCPE/GNP sensor

Identification of the specificity of the sensor was done for three electrodes. So, three separate electrodes were incubated with 1 μM of the RNA probe (for detection miR21) with 1 μM of the miR-21 and 1 μM of RNA (mismatch) and one electrode as bare which they incubated in the buffer for 24 h at 4 °C under similar experimental conditions. DPV and EIS techniques were used for monitoring the electrochemical reactions in PBS buffer (pH 7.4) in the presence of the [Fe(CN)_6_]^−3/−4^ redox couple. To evaluation the sensitivity of miR, increasing concentrations (1 a.M to 500 p.M) of miR21, were incubated with 1 μM of RNA probe in a 100 μL total volume of PBS at 37 °C for 1 h after incubation of 10 μL of the reaction mixture with cuco_2_o_4_/p19 sensor for 1 h at room temperature, and then washing 3 times with 500 μL of phosphate buffer (PB) and electrochemical reactions were done^16^. To examine the effect of the amount of p19 in limit of detection of 21miR, we dropped 0.4 µl of p19 on the sensor with the increasing concentrations (1 a.M to 500 p.M) as the same of conditions.

### Optimization of the cuco_2_o_4_/p19/ SCPE/GNP sensor

In this study, we determinate the electrochemical power of the cuco_2_o_4_ hallow structure in the sensor, and also the reproducibility of the sensor because of staying of p19 into the cuco_2_o_4_ structure by mimicking the native virus-like structure. To analysis the stability of this sensor, DPV was done in one electrode for 30 cycles, and the change of behavior was recorded.

### Cell culture and RNA extraction

We used as metastatic breast-cancer cell lines (MCF-7) to determine the miR-21 levels and Hella cell (a human cervix-adenocarcinoma cell line) as a control cell with no increase 21miR. MCF-7 is shown as estrogen-receptor-positive and Hella cell used as negative control cell lines, respectively. Cell lines including MCF-7, MCF-10A, Hella cells were cultured with 1% penicillin/streptomycin and growth in 5% CO_2_ at 37 °C. After collection of 10^7^ cells of conditioned medium, they centrifuged at 2000 × g for 30 min to eliminate cell debris. For RNA extraction of these cells, β-mercaptoethanol was used to the lysis to protect against the RNase activity. Then, we isolated the total RNA by purification kit (Norgen BioTek Corp., Canada) according to the protocol. The sample was divided two sample volumes of the lysis solution and 0.2 mL of the separation matrix. Next, we vortexed the mixture for 30 s, and incubated at 60 °C for 10 min. Three volumes of 99% ethanol were added to blending for 30 s. Subsequently, the mixture was centrifuged. The binding solution was then added to the pellet and incubated at 60 °C for 10 min. After adding 0.3 mL of 99% ethanol, the slurry was washed of the bound RNA to remove the remaining proteins and other impurities. The purified total miRNA was then eluted in 60 μL RNase free water^17^.

## Results

### Electrochemical behavior of the cuco_2_o_4_/p19/ SCPE/GNP sensor

**Figure 1:**
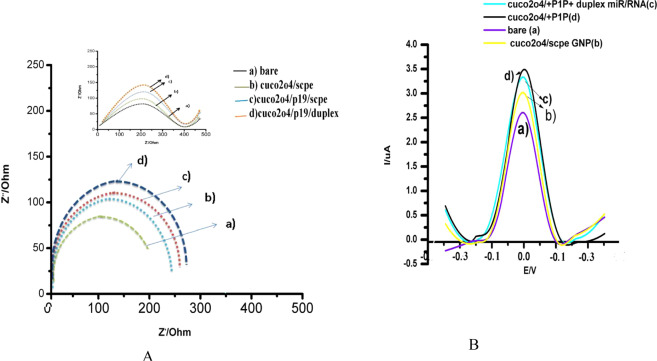
(**A**) EIS of behavior of the stages of cuco_2_o_4_/p19/Duplex + 21miR/ SCPE/GNP sensor for sensing 21miR.a) bare, b) cuco_2_o_4/_SCPE/GNP, c) cuco_2_o_4_/p19, d) cuco_2_o_4_/p19/Duplex + 21miR/ SCPE/GNP., (**B**) DPV of behavior of the stages of cuco_2_o_4_/p19/Duplex + 21miR/ SCPE/GNP sensor for sensing 21miR.a) bare, b) cuco_2_o_4/_SCPE/GNP, c) cuco_2_o_4_/p19/Duplex + 21miR/ SCPE/GNP, d) cuco_2_o_4_/p19. Modified surfaces recorded at a scan rate 50 mV s^−1^ in phosphate buffer (PH = 7.4) containing 1 mM [Fe(CN)6]^−3/−4^.

Cuco_2_o_4_ structure showed the different electrochemical behavior in presence of 1 mM [Fe(CN)_6_]^−3/−4^ redox probe in PBS buffer on the GNP-SCPE (Fig. 1A,B). Bare electrode showed the ΔE(Epa -Epc) 78 mV in the [Fe (CN)_6_]^−3/−4^ redox probe. The electrochemical behavior of cuco_2_o_4_ showed the peak currents decreased from 14.2 to 13.5 mA and the ΔEp increased from 78 to 81 mV, due to the facilitate electrolyte penetration into the interior surfaces by the nanoporous shells (Fig. 1B). This cuco_2_o_4_ structure can provide more active sites for electrochemical reactions by nano pores and a high specific surface area that they can facilitate the access of the electrolyte. In the previous studies, the addition of p19 protein to the substrates was via synthetic or non-synthetic linkers^18,27^. For example, in the ramnani’s research showed that the binding of p19 on the CNT/FET surface was via PBASE which it caused to reduce the resistance of the electrochemical substrate by covering the electrochemical reactions^28^. In this study, a virus-like cuco_2_o_4_ structure mimics of the intrinsic structure of CIRV. After adding p19 on this sensor, peak current increases from 13.5 to 14.1 mA and the ΔEp decreased from 81 to 70 mV (Fig. 1B). The interesting point of our study is the placement of the p19 into the cuco_2_o_4_ structure from pores by mimicking the CIR virus. In our study, the cuco_2_o_4_ pores were the hot-spot site for electrochemical exchanges between the internal and external charges. So the large amounts of p19s pass through these pores. The pH-induced in the core-shells can play a role to retain p19 in the space of shells. On the other hands, placement of p19 can inhibit the path of electron and decrease the resistance. The electrochemical behavior of the cuco_2_o_4_/p19/SCPE/GNP was examined by electrochemical impedance spectroscopy (EIS), which was an effective method for the characteristic of surface-modified electrodes^19,20^. Nyquist showed the electron transfer by impedance spectra include a semicircle portion. The model was used for this sensor is R (Q(RW))(QR). Figure 1(A) presents the Nyquist plot of (a) bare electrode, (b) cuco_2_o_4_/electrode, (c) cuco_2_o_4_/p19, (d) negative control sample in 10 mM [Fe(CN)_6_]^−3/−4^ and 0.1 M KCl at open circuit potential with frequencies varying from 0.1 Hz to 100 kHz. As shown in Fig. 1A (a), the bare electrode illustrated a R_*et*_ value of approximately 210 Ω. When cuco_2_o_4_ was added on the electrode, the R_*et*_ value increased by about 330 Ω (Fig. 1A(b)). R_*et*_ value was continuously increased to 380 Ω when the p10 protein was immobilized on the electrode surface. These results were in good agreement with that of differential pulse voltammetry.

### Model of sensing 21miR in the cuco_2_o_4_/ p19/SCPE/GNP sensor based on the virus-like structure

Adding duplex of 21miR/RNA on the cuco_2_o_4_/ p19/ SCPE/GNP showed the ΔE(Epa-Epc) 92 mV in the [Fe (CN)_6_]^−3/−4^ redox probe (Fig. 1B). The electrochemical behavior of cuco_2_o_4_ showed the peak currents decreased from 14.1 to 12.1 mA and the ΔEp increased and the Ret value increased by about 400Ω due to the stable interactions of duplexes of 21miR/RNA with p19, followed by the change of p19 conformational and enhancement the electrochemical reaction (Fig. 1A). Recent studies showed that the pH-dependent mechanisms can cause to maintain the proteins in the virus shell^30,31^. So, the micro RNA sensing mechanism can be explained by the virus-like model due to enhance the electron transfer of the change in p19 conformation with the redox probe [Fe(CN)_6_]^−3/−4^ to the electrode surface when it connected with the miR/RNA duplex. Since the resistance of the sensor in this stage is very high, it may become saturated p19 in every cuco_2_o_4_ structure. Therefore, it can connect with the duplexes of 21miR/RNA. Results of the unit value of the electrochemical tests were reported in the different stages in Table (s.1). On the hands, there are many cuco_2_o_4_ structures on the SCPE-GNP which they can behave as electrochemical reservists (Fig. 4).

**Figure 2:**
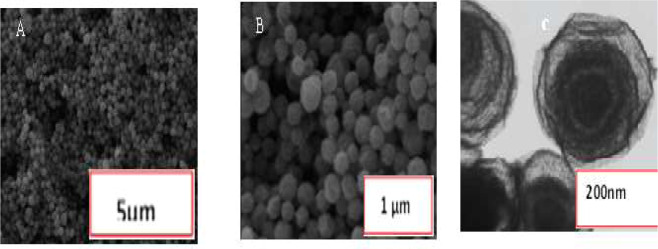
(**A**,**B**) FESEM and (**C**) TEM images of morphology of bimetallic (CuCo2O4). Figure A and B show (FESEM) Image of the Microspheres (CuCo2O4). The morphology and detailed structure of the samples were observed using the TESCAN Mira3 XMU field emisiion scanning electron microscope (FESEM) at accelerating voltage from 1-30 kV and Philips CM30 transmission electron microscope (TEM) at 300 Kv.

**Figure 3:**
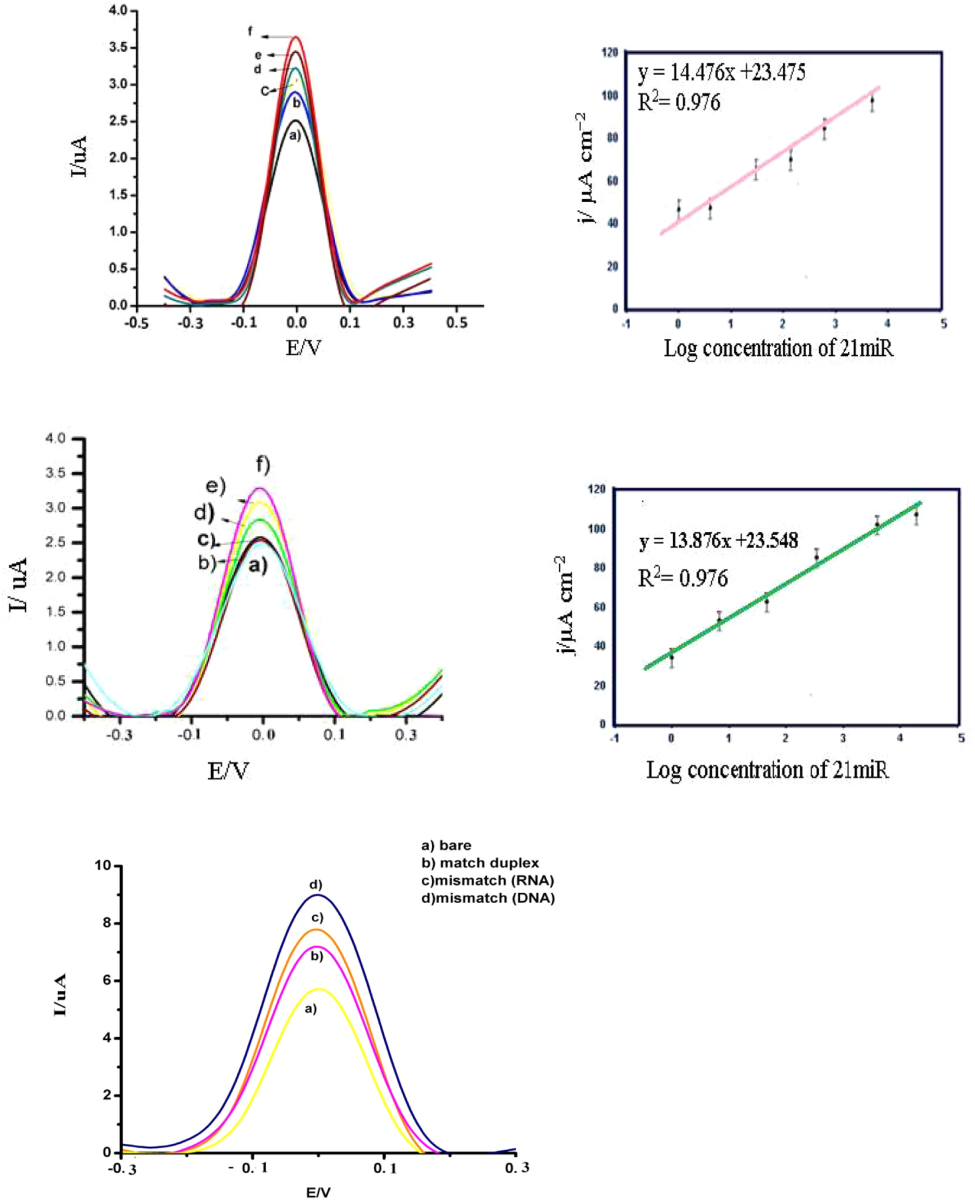
The performance of the sensor for detection of sensitivity and specificity using DPV measured at a scan rate 50 mV s-1 in phosphate buffer (pH = 7.4). (**A**) DPV of the cuco_2_o4/p19/ SCPE/GNP sensor with 0.1 µl P19, obtained using (f) 100 fM, (e) 1 fM, (d) 500 aM, (c)100 aM, (b) 10 aM, (a)1 aM of miR21 in the incubation buffer. (**B**) A calibration plot of the current density vs log concentration of miR-21 in DOPC-AuNP. (**C**) DPV of the cuco2o4/p19/ SCPE/GNP sensor with 0.5 µl P19obtained using (a) (f) 100 fM, (e) 1 fM, (d) 500 aM, (c)100 aM, (b) 10 aM, (a)1 aM of miR21 in the incubation buffer. (**D**) A calibration plot of the current density vs log concentration of miR-21 in DOPC/AuNP. (**E**) Specify of cuco2o4/p19/ SCPE/GNP sensor for detection complementary 21miR.

**Figure 4:**
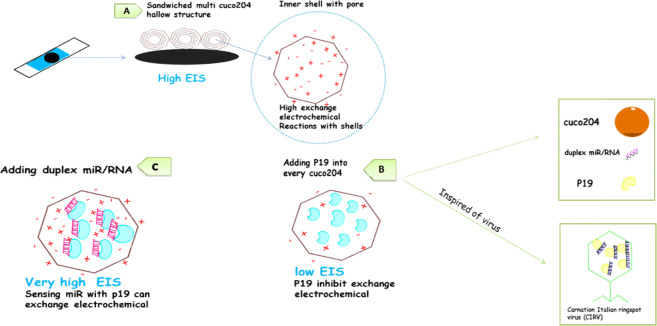
Schematic drawing of Detection of 21 miRs by the cuco2o4/p19/SCPE/GNP sensor mimicking CIR virus. (**A**) A fabrication of cuco2o4 hallow structure in the SPCE-GNP caused an increase in the resistance measured by EIS. (**B**) Adding p19 into the shells of cuco2o4 caused a decrease in the resistance measured by EIS. (**C**)Adding duplexes of 21miR/RNA caused an increase more resistance range.

### Surface morphology of the sandwiched cuco_2_o_4_ /p19 nanocomposite

The morphology of bimetallic (CuCo_2_O_4_) was examined by field-emission scanning electron microscopy (FESEM) and transmission electron microscopy (TEM). Figure 2A,B show (FESEM) image of the microspheres (CuCo_2_O_4_). It is visible that the samples have homogeneous microspheres morphology with a diameter of about 900 nm. The rough and wrinkle on the surface of the microspheres demonstrate a large-scale porous and the formation of a thin shell composed of small nanoparticles. As shown in Fig. 2C, TEM images of the microspheres, exhibit a multi-shelled structure with a uniform thickness around 20–30 nm. The results of TEM and FESEM about cuco_2_o_4_ /p19 nanocomposite also prove that there is no interaction of external factors on the surfaces was observed and no change about CuCo_2_O_4_ sizes (Fig s1).

### Identification of the specificity and sensitivity of the cuco_2_o_4_/p19/ SCPE/GNP sensor

In Fig. 3, the negative charge density was increased at the solution interface with the increasing electrostatic repulsion between the negatively charged of hybrids of miR21-probe and [Fe(CN)_6_]^−3/−4^. As above shown, when complementary hybrid formed, the peak currents (DPV) decreased and R_CT_ increased. In Fig. 3e, the negative control of hybridization with buffer with miR 21 showed an 32% increase in the j value (11.8132 μAcm^−2^) and attachment with non-complementary (target II) of 21 miR caused an 18.1%, increase in the j value (9.32 μAcm^−2^), respectively. DPV images of hybridization with mismatch RNA of miR21-probe indicated 22.9%, 22.32% increase in the j value (10.78 μAcm^−2^). The most important power of this study is the high diagnostic detection. Each nanocomposite structure of cuco_2_o_4_ can have a reservoir of p19 in the shell. In this study, we optimized the concentration of p19 (0.1 μl, 0.2 μl, 0.3 μl, 0.4 μl, 0.5 μl) to reaction with duplexes of 21mir at the same ranges (Fig s.2). The current concentration curve showed a linear relationship in the range of 0.1 μl to 0.5 μl of P19 concentration, with a sensitivity of 0.2 μl of 21miR (9033.4 μA mM^−1^ cm^−2^). Our result showed that in the least concentration of p19 (0.1 μl), there is a high detection of 21miR (Fig s.2 A, B). Results showed in Fig. 3A that the j value increases linearly with increasing the concentration of miR-21 ranged from 100 fM to 1aM when it was used as 0.1 μl p19. A regression equation of y = 14.476× + 23.475 (R^2^ = 0.976) was obtained, where y is the j value in μA cm^−2^ and x is the logarithmic concentration of 21miR in aM. As shown in Fig. 3B the relative standard deviation (RSD) values were between 9% and 0.1% and the limit of detection (LOD) was 1 aM. It was done from 3(Sb/m), where Sb is introduced as the standard deviation of the measurement signal for the blank and m detected the slope of the analytical curve in the linear region. On the other hand, with the concentration of 0.4 μl p19 at the same range of the concentration of miR-21(100 fM to 1aM), there is no change in the limit of detection (Fig. 3C,D). Results showed in Fig. 3C that the j value increases linearly with increasing the concentration of miR-21 ranged from 100 fM to1 aM when it was used as 0.4 μl p19. A regression equation of y = 13.876× + 23.548 (R^2^ = 0.976) was obtained, where y is the j value in μLA cm^−2^ and x is the logarithmic concentration of miR21 in aM. As shown in Fig. 3D the relative standard deviation (RSD) values were between 8% and 0.3% and the limit of detection (LOD) was 1 aM. The best reason for these results is the saturation of p19 in the low concentration with a duplex of 21miR/RNA. In the past research about p19-based biosensor, the limit of detections was the most of the time <1fM, when the concentrations of p19 were 0.5 μl^21–23^. These results may be due to the qualification of p19 connection with RNA/miR. Because of the p19 is stayed into the cuco_2_o_4_ structure in this model, the probability of the connection between the duplex and p19 is more than other p19-biosensors when they fix on the sensor (Table 1).

**Table 1:** Comparison of sensitivity of the different of liposomal substrates in electrochemical biosensor.

Lipid–platforms	Materials/linker	specificity	sensitivity	reproducibility	Complicated design	Ref
Electronic Detection of MicroRNA at Attomolar Level with High Specificity	CNTs-FET-p19/PBASE as linker in bed	++	++++++	+++++	+++++++	^26^
Magnetobiosensors Based on Viral Protein p19 for MicroRNA Determination in Cancer Cells and Tissues	Magnetic-p19 Not attach in bed	++	+++	+++	+++	^27^
Three-Mode Electrochemical Sensing of Ultralow MicroRNA Levels	AuNP/P19 Not attach in bed	+++	+++++++	++	+++++++++	^28^
Sequential or Multiplex Electrochemical Detection of miRs Based on the p19 Function Relative to three Sandwiches of Different Structural Hybrids on The Liposomal Sensor)	DOTAP-DOPE/p19 Not attach in bed	+++	++	++	+++++++++	^4^
exosomal bed by using multi covalent attachment p19	Esosome/p19 Attach in bed with no linker	++	+++++	+++++	++++	^4^
A new insight into electrochemical microRNA detection: A molec caliper, p19 protein	P19/ Not attach in bed	++	+++	++	++	^29^
A New Insight of Virus-like Hallow Structure in Electrochemical Biosensor to Detect an Attommolar of miR	P19/ preserved into the hallow structure	++	+++++	++++	+	This study

### Optimization of the reproducibility and stability of the cuco_2_o_4_/p19/ SCPE/GNP sensor

**Figure 5:**
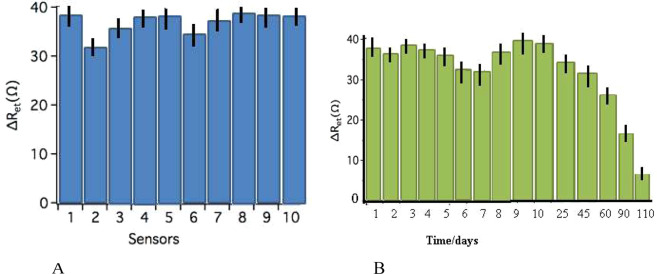
(**A**) Reproducibility image of DPV tests in cuco2o4/p19/ SCPE/GNP sensor bed with ten electrodes. These tests were done simultaneously on the separates electrodes. (**B**) DPV behaviors of stability of cuco2o4/p19/SCPE/GNP sensor sensors in different days. DPV show not too much change until 60 days.

We evaluate the reproducibility of the cuco_2_o_4_/p19/ miR/SCPE/GNP sensor with ten different modified sensors which were prepared to detect 0.1 nM of 21miR target sequence. As shown in Fig. 5(A), the results of relative standard deviation of measurements for the 10 repeats showed the 4.11%, which confirmed the high reproducibility for them. To detect the stability of the hallow structure biosensor, the cuco_2_o_4_/p19/ miR/SCPE/GNP sensor was first stored in the refrigerator at 4 °C for over 4 months and evaluated via EIS after its hybridization with 0.1 nM RNA/21miR sequence. In Fig. 5(B), we showed that there were the same signal occurred for 60 days. Afterward, the DPV signals was significantly decreased to 50.9.17%, and 90.2%, which corresponded to 90 days, 110 respectively. We haven’t seen the signal response when this sensor was stored for 111 days because the biological activity of the probe might be destroyed.

### Electrochemical detection of miRNAs in real samples

**Figure 6:**
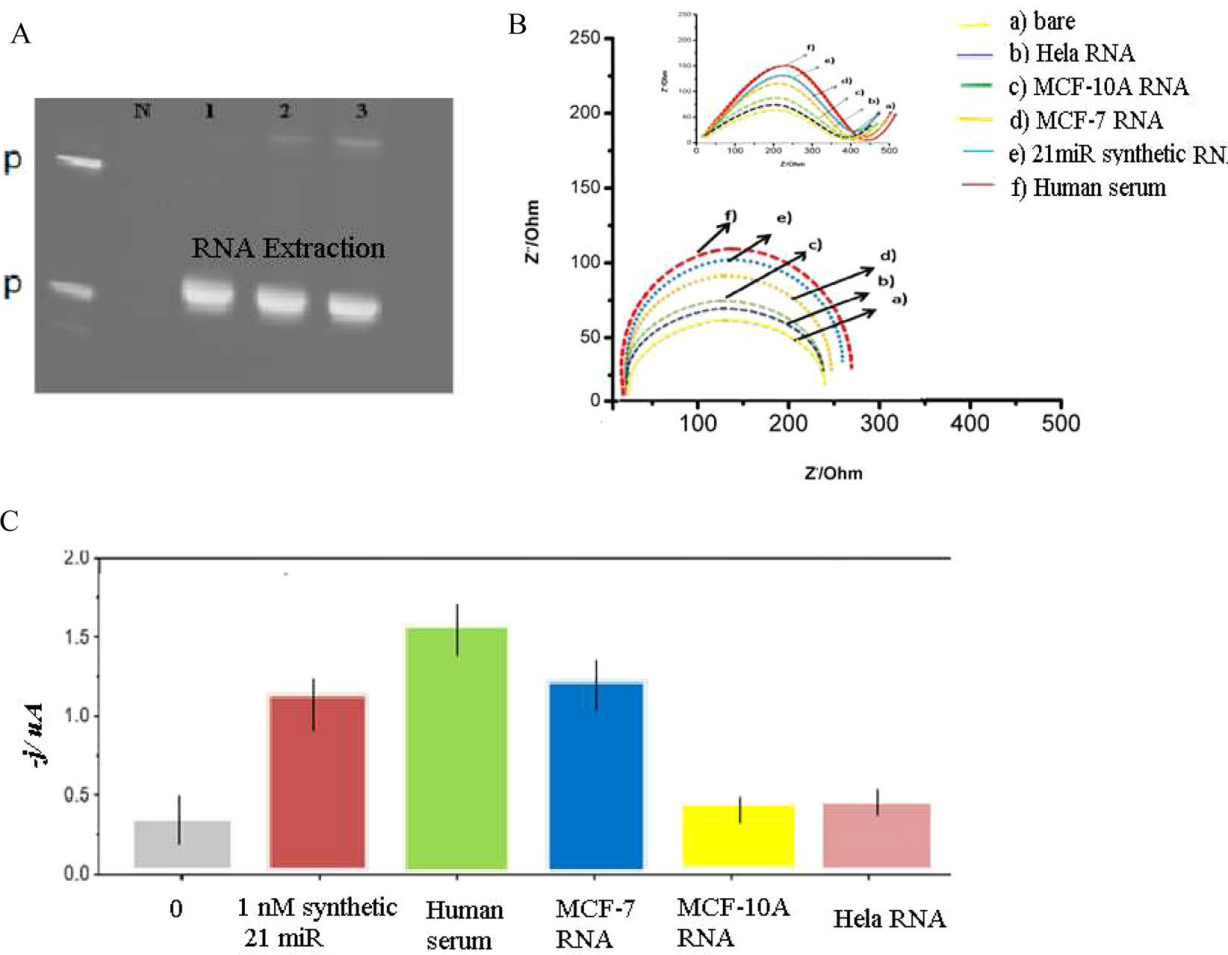
RNA extractions of cell lines and electrochemical detection of 21miR in the real-sampes. (**A**) RNA extraction of cell lines (1 = MCF-7, 2 = MCF-10A, 3 = Hela RNA). (**B**) EIS of behavior of the stages of cuco2o4/p19/ SCPE/GNP sensor for sensing 21miR in real samples. (**C**) Fig. 2. Differential chart of miR-21 in cell lines. Amperometric response measured with the cuco2o4/p19/ SCPE/GNP for RNAt (0.5 mg) isolated from different cell lines and the synthetic miR-21 target (10 fmol) used as a positive control. Error bars were estimated as triple the standard deviation (n = 3).

We used cuco_2_o_4_/p19/ SCPE/GNP to detect 21miRs in commercially human serum and MCF-7 / MCF-10A cells / HeLa cells. Before DPV experiments, an excess of yeast tRNA was added to a human serum sample to protect the endogenous miRNAs from nucleases. We used 1–5 mg of total raw RNA (RNA) isolated from these cells. The hallow structure biosensor response was significantly different for MCF-7 and MCF-10A cells, serum human RNA, thus indicating that miR-21 is highly expressed only in the metastatic cell lines (MCF-7), and not in the MCF-10A and HeLa cell lines (Fig. 6A). Based on the previous findings, miR-21 was overexpressed in MCF-7 cell line relate to the expression in the MCF-10A cell line^21,22^. On the other hand, we optimize the amount of RNA with amperometric signal. The best amount of extracted RNA from cells was about 0.5 mg (s. Figure 3). Our result obtained for five different samples as above described. RNA extracts were (22.1) fmol of miR-21 per microgram of RNA_t_ extracted from MCF-7 cells (RSD n = 5 = 12.6%). We also evaluated the discrimination efficiency towards the tested sequence 1 m by spiking RNAt (1.0 mg) extracted from MCF-10A cells and with the synthetic target (2 nm; ca. 18fmol) and 1 m oligonucleotides (2 nm; ca. 18 fmol) and MCF-7 cells RNA with the (17.4) fmol of miR-21 per microgram of RNA and 16.1 fmol of miR-21 per microgram of RNAt extracted from Hella cells. Results show the best signal detection of cuco_2_o_4_ biosensor is for human serum RNA > MCF-7 cells RNA > Synthetic 21 miR > MCF-10 cells RNA = Hela cells (Fig. 6C).The impedance spectra of the electrochemical tests were reported in the different cells and human cells are in Fig. 6B.

## Discussion

The large studies were researched about the design of the electrochemical sensors for identification micro RNAs^23,24^. The design of appropriate beds is important to provide a good response in the electrochemical reactions due to the low amount of miRs in the body fluids^25^. P19 protein is an important nanobiotechnology tool that increases the sensitivity of miR detection in the electrochemical sensors^26,27^.In the recent research, this protein is bound to the electrode via a linker on the electrode, so the probability of connection with the duplexes of RNA/miR is being challenged in every step^28,29^. So, p19-biosensors shows the low sensitivity especially in the real samples^30,31^.In our research we used as a structure which it prompt the sensitivity of p19. For the first time, we designed the virus-like hallow structure which it can increase the intensity of electrochemical reactions. The placement of various copper metals along with cobalt has provided many conductive and electrochemical properties. On the other hand, every hallow structure can mimic of virus-like CRIV and provide the reservoir of p19 proteins that they can connect with the more of duplex of RNA/miR without any linkers. Our sensor shows the best sensitivity (LOD = 1 aM) related to the sensitivity of other p19-biosensor^32–34^. On the hand, we evaluated this sensor in the real samples and we get the same acceptable results. These structures can open a new window for a wide range of medical purposes. As a result, the sensor has been able to detect miR with very high sensitivity and stability and high repeatability. In the next research, the other hallow structure should be studied as biosensor applications in the diagnosis and therapy.

## Supplementary information

Supplemental information.
