# Author Correction: Impediometric Electrochemical Sensor Based on The Inspiration of Carnation Italian Ringspot Virus Structure to Detect an Attommolar of miR

**DOI:** 10.1038/s41598-020-70343-0

**Published:** 2020-08-11

**Authors:** E. Ghazizadeh, Seyyed Ebrahim Moosavifard, Negin Daneshmand, Saeid kamari Kaverlavani

**Affiliations:** 1 Department of Medical Biotechnology, School of Medicine, Mashhad University of Medical Sciences, Mashhad, Iran; 2 Department of Advanced Medical Sciences & Technologies, School of Medicine, Jahrom University of Medical Sciences, Jahrom, 74148-46199 Iran; 3 Research Center for Noncommunicable Diseases, School of Medicine, Jahrom University of Medical Sciences, Jahrom, 74148-46199 Iran; 4 Department of Materials Science and Engineering, Shiraz university, Shiraz, Iran; 5 Department of Physics, Tarbiat Modares University, Tehran, Iran

Correction to: *Scientific Reports* https://doi.org/10.1038/s41598-020-66393-z, published online 15 June 2020

The original version of this Article contained errors in Affiliations 2 and 3, which were incorrectly given as ‘Department of Advanced Medical Sciences & Technologies, School of Medicine, Jahrom University of Medical Sciences (JUMS), Jahrom, 74148-46199, Iran’ and ‘Research Center for Noncommunicable Diseases, School of Medicine, Jahrom University of Medical Sciences (JUMS), Jahrom, 74148-46199, Iran’, respectively.

The correct affiliations are listed below:

Affiliation 2

Department of Advanced Medical Sciences & Technologies, School of Medicine, Jahrom University of Medical Sciences, Jahrom, 74148-46199, Iran.

Affiliation 3

Research Center for Noncommunicable Diseases, School of Medicine, Jahrom University of Medical Sciences, Jahrom, 74148-46199, Iran.

Additionally, in the Supplementary Information file originally published with this Article, Negin Daneshmand was omitted from the author list. Furthermore, the email address of E. Ghazizadeh was omitted.

These errors have now been corrected in the HTML and the PDF versions of the Article and in the accompanying Supplementary Information.

