# Supplementary material for: Impediometric Electrochemical Sensor Based on The Inspiration of Carnation Italian Ringspot Virus Structure to Detect an Attommolar of miR
Source: Sci Rep. 2020 Jun 15;10:9645. doi: 10.1038/s41598-020-66393-z (PMC7295965; doi:10.1038/s41598-020-66393-z)
Supplement: Supplementary file 1 — Supplemental information. [file 41598_2020_66393_MOESM1_ESM.docx]

**Impediometric Electrochemical Sensor Based on The**

**Inspiration of Carnation Italian Ringspot Virus Structure to Detect an Attommolar of miR**

**E. Ghazizadeh^1,^** ***, Seyyed Ebrahim Moosavifard ^2, 3,^** ***, Negin Daneshmand^4^, Saeid kamari kaverlavani^5^**

**^1^Department of Medical Biotechnology, School of Medicine, Mashhad University of Medical Sciences, Mashhad, Iran**

**^2^Department of Advanced Medical Sciences & Technologies, School of Medicine, Jahrom University of Medical Sciences, Jahrom 74148-46199, Iran**

**^3^Research Center for Noncommunicable Diseases, School of Medicine, Jahrom University of Medical Sciences, Jahrom 74148-46199, Iran**

**^4^Department of Materials Science and Engineering, Shiraz University, Shiraz, Iran**

**^5^Department of Physics, Tarbiat Modares University, Tehran, Iran**

* **elhamgenetic@yahoo.com; info_seyyed@yahoo.com**

Table 1s. Results of the unit value of the electrochemical tests at the different stages of the sensor

| **Adding**  **Duplexes of 21miR/RNA** | **Adding**  **P19 on the**  **cuco2o4/SCPE-GNP** | **Sandwiched**  **cuco2o4/SCPE-GNP** | **step** |
| --- | --- | --- | --- |
| 92 | 85 | 78 | ***ΔEp***  **mV))** |
| 1.93×10^4^ | 1.74×10^4^ | 1.98×10^4^ | ***R_CT_* Ω (cm^-2^*)*** |


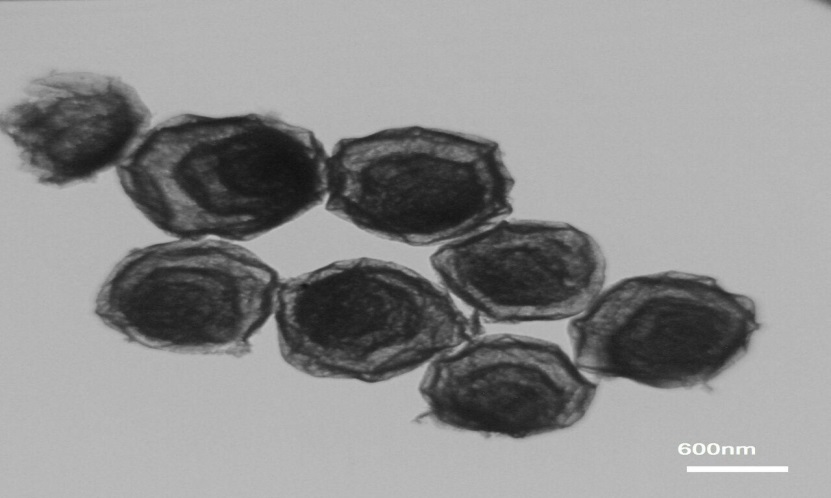


Fig. 1s. TEM images of morphology of CuCo2O4/p19 as control image. As shown in figure, TEM images of the CuCo2O4/p19 stage, exhibit a multi-shelled structure with a no extra materials on the structure. It means p19 permeate of 20 nm pores and placed into the shell.

b)

a)


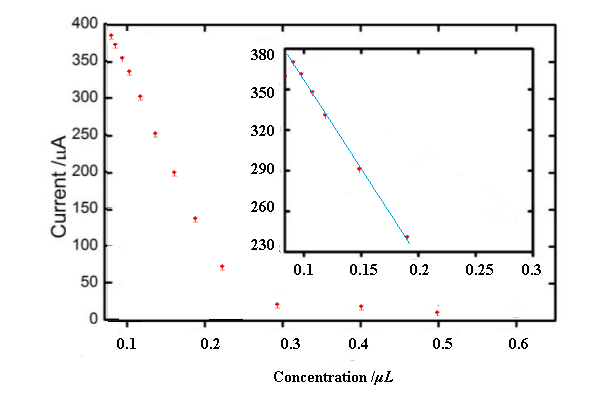

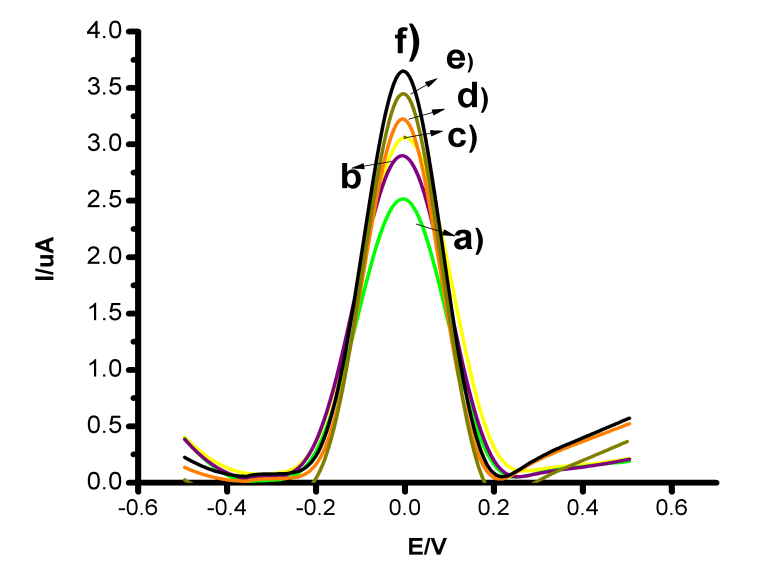
Fig. 2s. The effect of concentration of p19 to detection of 21miR. (a) differential pulse voltammetry (DPV) curves of the cuco2o4/SCPE-GNP electrode containing b) 0.1,c) 0.2, d) 0,3, e) 0,4, f) 0.5 μl p19 to detection 0.2 μl miR21, at a scan rate of 0.1 V s−1; (b) The calibration curves of the biosensor.


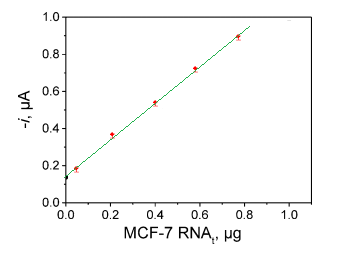


Fig. 3s. Correlation of the amperometric response on the quantity of RNA extracted from MCF-7 cells. Error bars were estimated as triple the standard deviation (n=3)
